# mRNA-based vaccines synergize with radiation therapy to eradicate established tumors

**DOI:** 10.1186/1748-717X-9-180

**Published:** 2014-08-15

**Authors:** Mariola Fotin-Mleczek, Kai Zanzinger, Regina Heidenreich, Christina Lorenz, Aleksandra Kowalczyk, Karl-Josef Kallen, Stephan M Huber

**Affiliations:** CureVac GmbH, CureVac GmbH, Paul-Ehrlich-Str. 15, Tübingen, 72076 Germany; Department of Radiation Oncology, University of Tübingen, Tübingen, Germany

**Keywords:** mRNA-based vaccines, Immunoradiotherapy, Immunotherapy, Radiotherapy, Lewis lung carcinoma, E.G7-OVA

## Abstract

**Background:**

The eradication of large, established tumors by active immunotherapy is a major challenge because of the numerous cancer evasion mechanisms that exist. This study aimed to establish a novel combination therapy consisting of messenger RNA (mRNA)-based cancer vaccines and radiation, which would facilitate the effective treatment of established tumors with aggressive growth kinetics.

**Methods:**

The combination of a tumor-specific mRNA-based vaccination with radiation was tested in two syngeneic tumor models, a highly immunogenic E.G7-OVA and a low immunogenic Lewis lung cancer (LLC). The molecular mechanism induced by the combination therapy was evaluated via gene expression arrays as well as flow cytometry analyses of tumor infiltrating cells.

**Results:**

In both tumor models we demonstrated that a combination of mRNA-based immunotherapy with radiation results in a strong synergistic anti-tumor effect. This was manifested as either complete tumor eradication or delay in tumor growth. Gene expression analysis of mouse tumors revealed a variety of substantial changes at the tumor site following radiation. Genes associated with antigen presentation, infiltration of immune cells, adhesion, and activation of the innate immune system were upregulated. A combination of radiation and immunotherapy induced significant downregulation of tumor associated factors and upregulation of tumor suppressors. Moreover, combination therapy significantly increased CD4^+^, CD8^+^ and NKT cell infiltration of mouse tumors.

**Conclusion:**

Our data provide a scientific rationale for combining immunotherapy with radiation and provide a basis for the development of more potent anti-cancer therapies.

**Electronic supplementary material:**

The online version of this article (doi:10.1186/1748-717X-9-180) contains supplementary material, which is available to authorized users.

## Introduction

Despite recent advances in the field of cancer immunotherapy, the treatment of large, advanced tumors remains very challenging and the clinical outcome is often disappointing [1, 2]. There are some encouraging clinical results, however, which show that combining targeted therapies (e.g. EGFR inhibitors, anti-angiogenic agents, vaccines) with local radiation can result in an improved clinical outcome. Because of our experience in mRNA-based cancer vaccine development we sought to determine if mRNA immunotherapy and local tumor radiation could act synergistically to inhibit the growth of large, established tumors. Next, we asked if combined immunoradiotherapy could also inhibit the growth of low immunogenic tumors such as Lewis lung cancer (LLC). Finally, to get an insight into the mechanisms involved, we looked at cellular and molecular changes at the tumor sites following combination therapy.

Different strategies for combined anti-tumor therapy have already been tested. Among these approaches, which include chemotherapy, small molecule inhibitors and monoclonal antibodies, radiation therapy is the most interesting candidate for combining with active immunotherapy. Not only can radiation destroy tumor cells, it can also induce substantial changes in the tumor microenvironment. The effects of radiation differ over time and two types of responses (immediate and delayed) have been characterized. The immediate responses are mostly limited to the rapid protein degradation, generation of novel peptide repertoires and upregulation of MHC class I expression [3]. The delayed response consists of increased protein synthesis, induction of Fas signaling, upregulation of costimulatory molecules and induction of adhesion molecules [4]. These changes at the radiated tumor site can significantly affect the action of immune cells which are already present or which will be recruited to the tumor site following immunotherapy. The main challenge for an efficacious combination therapy is the determination of the optimum dose and schedule which should result in a synergistic effect and avoid negative events such as killing of the radiosensitive immune cells. Because the majority of the radiation induced immunological changes at the tumor site are only transient it would be desirable to combine radiation with immunotherapy to induce sustained memory responses against different antigens and to mediate long-lasting tumor protection. Moreover, while radiation therapy is successfully used to treat single, small and spatially well-defined tumors, it is not suitable for the systemic treatment of non-symptomatic metastatic disease. Thus, combining radiation therapy with immunotherapy, which induces systemic responses, should be advantageous.

We have previously shown that mRNA-based two-component cancer vaccines consisting of free and complexed mRNA induce sustained, balanced immune responses and mediate strong tumor protection *in vivo*, thus represent a novel promising approach to cancer therapy [5]. Here, we demonstrate that both therapies (systemic mRNA immunotherapy and local tumor irradiation) act synergistically to eradicate established macroscopic E.G7-OVA and LLC tumors. Moreover, the molecular and cellular analyses of the changes that occur at the tumor sites give us an insight into the mechanisms involved. Our data demonstrate that the induction of mechanistic changes in the tumor microenvironment, combined with a potent immunotherapy, may lead to the significant advances in the fight against cancer.

## Material and methods

### Material

Protamine was obtained from Valeant Pharmaceuticals Germany GmbH (Germany). Following antibodies were purchased from eBioscience (Germany): anti-NK1.1-PerCP-Cy5.5 (clone PK136), anti-CD4-APC (clone RM4-5), anti-CD11c-APC (clone N418), anti-CD8-PE-Cy7 (clone 53-6.7) and anti-F4/80-APC-eFluor780 (clone BM8). Anti-CD11b-PE-Cy7 (clone M1/70), anti-CD3-APC-Cy7 (clone 145-2C11) and anti-CD45.2-HV450 (clone 104) were obtained from BD Bioscience (Germany). SIINFEKL and control peptides were purchased from Bachem AG (Germany). Collagenase type IV was obtained from Sigma-Aldrich (Germany) and DNAse I from Roche (Germany).

### RNActive^®^ technology

CureVac GmbH proprietary technology generates mRNA molecules with increased stability and translatability (claimed in the patents: EP 1392341 and EP 1604688). All mRNA vaccines used in this study were produced according to this technology.

### Two-component mRNA vaccine

mRNA was protamine-formulated as described previously [5]. Briefly, the vaccine consists of a mixture of 50% free mRNA (component 1) and 50% mRNA complexed with protamine at a weight ratio of 2:1 (component 2). First, mRNA was complexed by addition of protamine-Ringer lactate solution and after stable complexation free mRNA was added.

### Mice and cell lines

C57BL/6 mice (7 - 9 weeks old) were purchased from Janvier Laboratories (France). All experimental procedures were performed in compliance with protocols approved by the commission of the Regierungspraesidium Tuebingen. EL-4 T cell lymphoma and E.G7-OVA, a mouse T cell lymphoma cell line stably expressing *Gallus gallus* Ovalbumin (GgOVA), were purchased from LGC Promochem GmbH (Germany). A mouse Lewis lung carcinoma (3LL-GFP cell line stably expressing *Aequorea victoria* green fluorescence protein (GFP)), was a gift from B. Wielockx, University Hospital/Faculty of Medicine Carl Gustav Carus at the Technische Universität Dresden.

### Tumor challenge

Mice were transplanted subcutaneously (s.c.) with either E.G7-OVA (3×10^5^ or 5×10^5^) cells or 3LL-GFP (5x10^5^) cells for therapeutic vaccination and 1×10^6^ E.G7-OVA for prophylactic vaccination. Tumor growth was monitored over time by measuring tumor size in 3 dimensions using calipers. Tumor volume was calculated using following formula: Tumor volume (mm^3^) = (length (mm) × π × width^2^ (mm^2^))/6.

### Vaccination

For prophylactic vaccination, mice were anesthetized and vaccinated twice with 7-day interval. Animals were challenged one week later. For therapeutic vaccination, mice were inoculated with tumor cells and left untreated till the tumor reached the volume indicated in the Figure legends. At that time, mice were anesthetized and injected with mRNA vaccine (8 or 16 μg per injection site as indicated; 4 injection sites/mouse distributed at the lower and upper back). Untreated animals served as a control.

### Irradiation

For radiation experiments, mice were transplanted s.c. with tumor cells into the right hind limb and tumors were locally X-ray-irradiated under isoflurane anesthesia at room temperature using a linear accelerator (LINAC SL25, Phillips) with a dose rate of 4 Gy/min. Local irradiation of tumors was performed on 3-4 consecutive days with different doses with or without combination with mRNA vaccination as stated in the Figure legends. Only tumor site was irradiated, the remaining body parts, including vaccination sites, were covered by a shield.

### qRT-PCR

Total RNA was extracted from explanted tumors using the Qiagen RNeasy Mini Kit according to the manufacturer’s instruction. Expression levels of GgOVA were quantified via RT-PCR by Biolytix AG (Switzerlad), using mGAPDH as a reference gene. All reactions were performed in triplicates.

### Enzyme-linked immunosorbent spot

Splenocytes were isolated 6 days after the last vaccination. Splenocytes from mRNA-vaccinated and control mice were stimulated with 1 μg/ml of either relevant (SIINFEKL for OVA-vaccinated or EGFR-peptide library for EGFR-vaccinated mice) or control MHC class I restricted peptides. Secreted IFNγ was detected using a standard ELISpot protocol and measured using a plate reader (Immunospot Analyzer, CTL Analyzers LLC).

### Microarray analysis

20 days after tumor challenge mice were euthanized; complete tumors were excised and stored in RNAlater RNA Stabilization Reagent (QIAGEN, Germany). Total RNA was extracted by homogenization with RNeasy isolation kit (QIAGEN, Germany) according to the manufacturer’s protocol. 10 μg purified RNA were used for microarray analysis. RNA integrity and quantity was evaluated on Bioanalyzer-2100 (Agilent Technologies). Gene expression analysis was performed by MFTServices (Tübingen, Germany) via whole-genome RNA microarray (Affymetrix, UK) and analyzed with Ingenuity IPA Software (INGENUITY Systems, USA).

### Flow cytometic analysis

For flow cytometric analysis of tumor infiltrating immune cells, mice were euthanized, complete Lewis Lung tumors were excised and a single cell suspension was generated via 30 min collagenase digestion at 37°C followed by tissue dissection with the gentleMACS (Miltenyi Biotech, Germany). Single cell suspension was stained for CD45.2 to distinguish between CD45.2^-^ tumor cells and CD45.2^+^ host-derived immune cells. The following immune cell populations were further characterized: CD3^+^CD4^+^ T helper cells, CD3^+^ CD8^+^ cytotoxic T lymphocytes, CD3^+^ NK1.1^+^ NKT cells, CD3^-^ NK1.1^+^ NK cells, CD11c^+^ dendritic cells, CD11b^+^ F4/80^+/-^ myeloid cells. Data were analyzed using FlowJo software (TreeStar, USA).

### Statistical analysis

Statistical analysis was performed using GraphPad Prism software, Version 5.01. The differences between groups were analyzed using *t*-test or Mann-Whitney test, depending on the data distribution. Tumor growth was shown as mean ± SEM. Analysis of grouped data was performed using 2-way ANOVA with Bonferroni posttests. Survival curves were analyzed using Log-rank (Mantel-Cox) Test.

## Results

### mRNA-based cancer vaccines induce high frequencies of antigen-specific T cells and mediate tumor protection during prophylactic and therapeutic treatment

**Figure 1:**
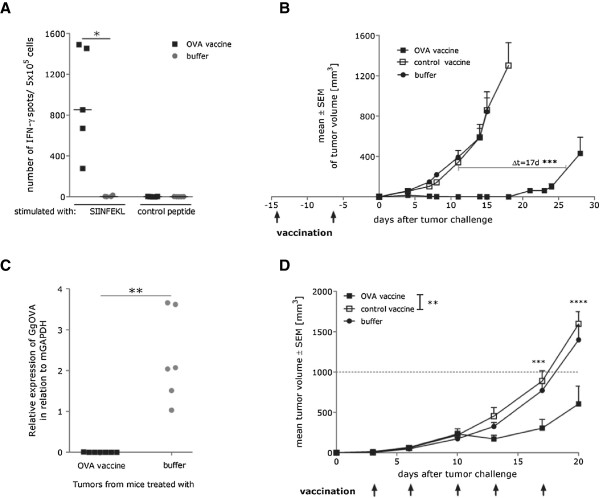
**Efficacy of RNA immunotherapy is strongly dependent on the tumor burden and time available for the induction of immune response. (A)** C57BL/6 mice (n = 5 per group) were vaccinated 2 times (1 vaccination/week) either with OVA mRNA vaccine (32 μg) or buffer. After 7 days splenocytes from vaccinated mice were analyzed for IFN-γ secretion in response to Ovalbumin- or Connexin-derived epitope using an ELISpot assay. * - p = 0.0154 **(B)** C57BL/6 mice (n = 8 per group) were vaccinated 2 times (1 vaccination/week) either with OVA mRNA vaccine (64 μg), control vaccine (64 μg) or buffer. 6 days after the second vaccination, mice were challenged subcutaneously with 1 × 10^6^ syngenic E.G7-OVA tumor cells. Tumor growth was monitored by measuring the tumor size in 3 dimensions using calipers. *** - p < 0.0001 **(C)** Expression of Ovalbumin in tumors escaping the control of the immune system, following prophylactic vaccination, was analyzed. Total RNA was isolated and OVA expression was quantified via qRT-PCR in relation to mGAPDH. ** - p = 0.0034 **(D)** C57BL/6 mice (n = 8 per group) were challenged s.c. with 0.3 × 10^6^ syngenic E.G7-OVA tumor cells on day 0. On day 3 mice were treated either with OVA vaccine (32 μg), control vaccine (32 μg) or buffer. Tumor growth was monitored by measuring the tumor size in 3 dimensions using calipers. ** - p = 0.0091, ***- p < 0.001, ****- p < 0.0001. All presented data show representative results of at least two independent experiments.

To demonstrate the potential of two-component mRNA-based cancer vaccines to mount strong immune responses, we vaccinated mice twice with a vaccine encoding the model antigen ovalbumin (OVA). Six days after the boost vaccination, high frequencies of OVA-specific T cells were detected in the splenocytes of vaccinated mice (Figure 1A). Moreover, prophylactic immunization with the OVA-encoding vaccine significantly delayed the growth of E.G7-OVA tumors (Figure 1B). Interestingly, qRT-PCR analysis of escaping tumors revealed a complete loss of OVA expression in the recurring tumors. These data suggest that the vaccination enhanced tumor surveillance resulting in the tumor outgrowth due to either antigen loss or the selection of OVA-negative cells (Figure 1C). Next, we tested the efficacy of the mRNA-based OVA vaccine in treating small, established tumors. We showed that despite the very limited time window for the induction of the immune response, frequent vaccination with two injections per week was able to induce a statistically significant delay in tumor growth compared to control animals (p = 0.0091) (Figure 1D). However, the effect was less pronounced compared to the prophylactic treatment. In summary, our results show that our two-component mRNA vaccine induces strong protective immunity and delays tumor growth in therapeutic settings.

### mRNA vaccination and radiation therapy act synergistically to facilitate the eradication of large established tumors and mediate protection against re-challenge with parental tumor cells

**Figure 2:**
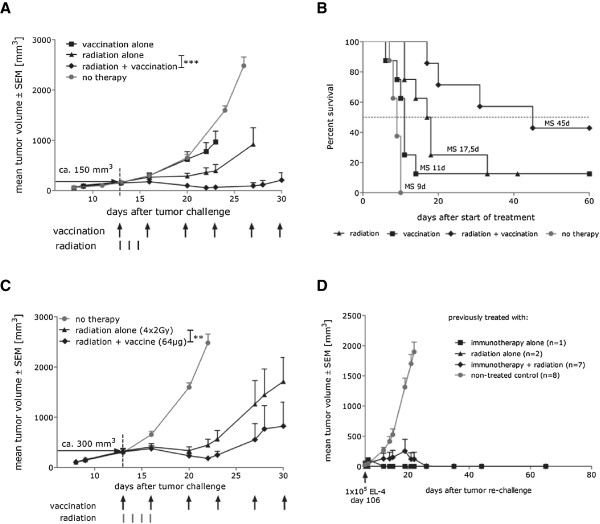
**Combination of RNA immunotherapy with radiation allows for the eradication of large established tumors and induction of epitope spreading. (A)** C57BL/6 mice (n = 8 per group) were challenged subcutaneously on the right limb with 0.3 × 10^6^ syngenic E.G7-OVA tumor cells. 13 days after tumor challenge (at a median tumor volume of 150 mm^3^) mice were treated either with OVA mRNA vaccine (32 μg), radiation (6 Gy total, divided into 3 equal fractions on 3 consecutive days) or radioimmunotherapy as indicated (with first vaccination given 6h before first radiation). Untreated mice served as a control. *** - p < 0.001 **(B)** Median survival time of mice analysed in Figure 2A. **(C)** C57BL/6 mice (n = 8 per group) were challenged subcutaneously on the right limb with 0.5 × 10^6^ syngenic E.G7-OVA tumor cells. 13 days after tumor challenge (at a median tumor volume of 300 mm^3^) mice were treated either with local radiation (8 Gy total, divided into 4 equal fractions on 4 consecutive days) or radioimmunotherapy as indicated (64 μg/vaccination, with first vaccination given 6 h before first radiation). Untreated mice served as a control. ** - p < 0.01 **(D)** All complete responders, which were tumor free after combination therapy (day 106), were re-challenge subcutaneously with 1 × 10^5^ parenteral OVA-negative EL-4 cells. Tumor growth was monitored by measuring the tumor size in 3 dimensions using calipers. All presented data show representative results of at least two independent experiments.

Therapeutic vaccines alone are not effective in curing bulky, large tumors due to the time required to induce a potent immune response as well as tumor-mediated inhibitory mechanisms. We, therefore, asked whether the combination of RNA immunotherapy with local irradiation could result in the successful treatment of the established tumors. E.G7-OVA tumor cells were transplanted subcutaneously into the right limb and mice were left untreated until the tumor reached a volume of about 150 mm^3^. Since immunotherapy and radiation therapy can successfully treat high percentage of small tumors, the large tumor size as well as the delayed start of the therapy was chosen to enable demonstration of the synergistic effect of radioimmunotherapy in the settings when neither of the monotherapies was sufficient. Tumors of mice in the radiation and the combined radioimmunotherapy groups were irradiated with a total dose of 6 Gy, administered in 3 equal fractions for three consecutive days. Mice in the immunotherapy and the radioimmunotherapy groups received two vaccinations per week starting at the first day of radiation. Immunotherapy alone showed only a marginal effect on tumor growth (Figure 2A), which was expected due to the limited time during which an immune response can develop while tumors are in the exponential growth phase. Similarly, radiation alone induced only a transient inhibition of tumor growth for about 7 days. In contrast, combined radioimmunotherapy significantly improved the anti-tumor effect (Figure 2A). All mice treated with this regimen showed pronounced tumor regression, resulting in complete and sustained eradication of the tumor in 3 out of 7 mice. Median survival in the combination group increased significantly to 45 days after the start of treatment, compared to 9 days for untreated mice (p = 0.0002), 11 days for mice receiving immunotherapy (p = 0.0176) and 17.5 days for mice receiving radiation therapy (p = 0.045) (Figure 2B).

The efficacy of tumor treatment strongly depends on the initial tumor burden. Therefore, to further determine the potency of radioimmunotherapy in the next experiment we challenged mice with a higher number of tumor cells and investigated the effect of combination therapy on larger tumors with a volume of about 300 mm^3^. A group receiving vaccination alone was not included in this study because we know that vaccination is not effective in mice with a high tumor burden. We increased the total radiation dose to 8 Gy which was administrated in four equal fractions for four consecutive days and increased the dose of the OVA vaccine to 64 μg per vaccination. Mice were vaccinated twice per week. We demonstrated that combined radioimmunotherapy induced a strong synergistic anti-tumor effect, resulting in significant tumor shrinkage when compared to radiation treatment alone (Figure 2C). Moreover, although both monotherapies resulted in anecdotal complete responders, there was a high frequency of surviving animals in the radioimmunotherapy treated mice suggesting induction of a more effective immune response within this group. To further characterize the potency of the radioimmunotherapy we challenged the surviving mice with the parental, OVA-negative EL4 tumor cells. As shown in Figure 2D majority of the complete responders from radioimmunotherapy treated group (5/7) survived the subsequent challenge with parental tumor cells. Taken together, our data indicate that combined radioimmunotherapy results in stronger tumor growth retardation and greater survival compared to each treatment modality alone.

### Combined radioimmunotherapy is effective against low immunogenic, radiation-resistant Lewis lung cancer

**Figure 3:**
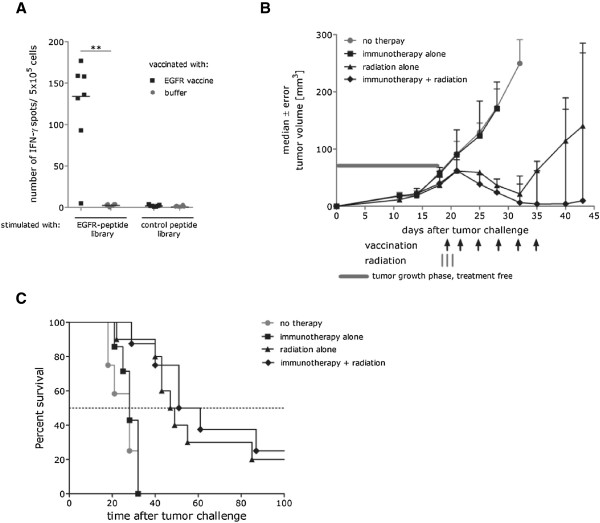
**Combination of RNA immunotherapy with radiation represents an effective treatment strategy for low immunogenic and radioresistant LLC tumor. (A)** C57BL/6 mice (n = 5 per group) were vaccinated 2 times (1 vaccination/week) either with EGFR mRNA vaccine (32 μg) or with buffer. 7 days after completed vaccination splenocytes from vaccinated mice were analyzed for IFN-γ secretion in response to EGFR- or Control-peptide library using an ELISpot assay. ** - p = 0.0036 **(B)** C57BL/6 mice (n = 10 per group) were challenged subcutaneously on the right limb with 5 × 10^5^ 3LL-GFP cells. 18 days after tumor challenge mice were treated either with local radiation (36 Gy total, divided into 3 equal fractions on 3 consecutive days) or with radioimmunotherapy as indicated (with first vaccination given 6 h before second radiation). Vaccination therapy without radiation started on day 14 post tumor challenge. Untreated mice served as a control. Tumor growth was monitored by measuring the tumor size in 3 dimensions using calipers. **(C)** Median survival time of mice analyzed in Figure 3B.

Having demonstrated the strong synergistic effect of radiotherapy and mRNA immunotherapy in the treatment of highly immunogenic E.G7-OVA tumors, we sought to determine whether this therapeutic strategy is also effective against one of the most challenging tumor models: LLC. This tumor model is resistant to different kinds of therapeutic regimens (Avastin, radiation, adoptive T cell transfer) [6–8]. It is characterized by nearly complete MHC class I down regulation, a lack of known tumor rejection antigens, very aggressive growth kinetics, frequent ulceration in small tumors and rapid formation of spontaneous lung metastases from the primary solid tumor. We chose human EGFR and Connexin as target antigens because of the evidence for their immunogenicity [9]. As shown in Figure 3A, application of the EGFR mRNA vaccine (5 vaccinations in total, twice a week, 32 μg per vaccination) resulted in a strong induction of EGFR-specific T cell responses compared to control animals (p = 0.0036). In corroboration with the literature data [10] dose finding studies revealed that LLC is strongly resistant to radiation (Additional file 1: Figure S1). As shown in Figure 3B, to achieve a weak and transient inhibitory effect on tumors with a volume of just 50 mm^3^, a total dose of 36 Gy was required. This dose was split into three fractions, given on consecutive days, starting on day 18 after tumor challenge. As shown in Figure 3B, immunotherapy alone was not able to mediate any tumor growth inhibition and radiation therapy alone resulted in only transient inhibition. In sharp contrast, mRNA vaccines combined with a high dose of radiotherapy resulted in a strong synergistic anti-tumor effect. In conclusion, our data show the efficacy of radioimmunotherapy in controlling tumor growth of a low immunogenic carcinoma.

### Radiation, immunotherapy and combined radioimmunotherapy induce distinct changes in gene expression at the tumor site

Immunotherapy or radiation therapy alone may be insufficient to eliminate bulky tumors. However, our data demonstrate the potency of a combined treatment. To elucidate the mechanisms responsible for the synergistic effects of RNA vaccines and local radiation, we first compared the gene expression profiles from non-treated control tumors and tumors from mice treated with either immunotherapy or radiation alone. Additionally to gain a mechanistic insight into the effect of radiation on immunotherapy we performed analyses to determine the changes in gene expression following combination therapy in comparison to vaccination alone. Mice were challenged with E.G7-OVA cells and were left untreated till tumors reached a volume of about 150 mm^3^. Subsequently mice were treated with either radiation at a total dose of 6 Gy applied for three consecutive days or/and OVA-encoded mRNA vaccine. Seven days after the start of treatment, 4 tumors per group were excised, total RNA was extracted and gene expression analyzed. Differentially expressed genes were identified by statistical analysis with the p < 0.05 and a fold change of at least 1.5.

To exclude the effect of distinct tumor burdens, tumors with similar volumes from the treated groups were chosen for analyses. Despite comparable volumes, tumors exhibited distinct gene expression profiles allowing for the correct segregation of the single samples into the corresponding treatment groups. As presented in Table 1 the analysis revealed that a broad panel of genes, associated with specific mechanistic pathways, was upregulated in the radiated tumors. The most prominent functional pathways upregulated after radiation were associated with angiogenesis, adhesion, chemotaxis, antigen presentation, T cell and NK signaling as well as with activation of the complement system and Toll-like receptors. Interestingly, immunotherapy alone also resulted in a pronounced change at the tumor site. We detected a high number of upregulated genes associated with T cell, NK cell, TLR signaling, chemotaxis, antigen presentation and activation of the complement system (Table 2). To elucidate the molecular mechanism responsible for the synergistic effects of the combination therapy we analyzed the gene expression in the radiation and immunotherapy-treated mice compared to the mice receiving vaccination only. Interestingly, this analysis revealed that high number of differentially regulated genes was downregulated (Table 3). Many of these genes encoded for tumor associated factors or molecules connected with tumor invasiveness and progression such as matrix metallopeptidases. In contrast, a number of genes described as tumor suppressors (among them osteoglycin, dermatopontin and secreted frizzled-related protein 4) were significantly upregulated after combination therapy. In conclusion, our data show induction of a unique gene signature following radioimmunotherapy, which is distinct from radiation or vaccination treatment alone, indicating a qualitative difference in the combined therapy.

**Table 1:** **Genes upregulated upon radiation**

Pathway/function	Genes upregulated upon tumor radiation
T cell signaling	Cd8b1 (2.8), Cd8a (2.6), Il12r (2.2), Il2r (2.1), Gzmb (3.8), Gzmk (1.9), Prf1 (2.2), Icos (2.4), Ctla4 (1.4), Eomes (1.6), Stat1 (1.6), Stat4 (2.4)
NK cell signaling	Klrd1 (3.1), Klrk1 (2.1), Klrc1 (3.0), Klra2 (1.6), Nkg7 (4.0), Il12r (2.2), Gzmb (3.8), Gzmk (1.9), Prf1 (2.2), Fcgr4 (2.2)
Chemotaxis	Ccl3 (2.0), Ccl5 (2.5), Ccl8 (2.1), Ccl11 (2.2), Cxcl12 (1.6), Cxcl16 (2.1), Ccrl2 (2.1), Ccr5 (1.7), Cxcr3 (1.5)
TLR signaling	Tlr7 (1.9), Tlr8 (2.2), Tlr9 (1.6), Tlr13 (1.7), Irak3 (1.5), Aebp1 (2.5)
MHC class II presentation	H2-Dma (1.8), H2-Ab1 (1.7), H2-Eb1 (1.5), H2-Aa (1.9), H2-T24 (1.8), Cd74 (1.7), Cathepsins: k, s, c, h, w, a (1.6-2.0), Anpep (1.9)
Complement system	C1qa (2.0), C1qb (1.6), C1qc (2.1), C1r (1.7), C2 (2.2), C3 (2.0), C4b (2.6), Cfb (2.1), Cfh (1.9)
Dendritic cells, macrophages	Cd11c (Itgax) (1.9), Cd11b (Itgam) (1.7), Clec4a1 (2.1), Clec4a3 (2.2), Ly6i (2.3), Sema4 (1.9), Blnk (1.6), Lrp1 (2.6), Aebp1 (2.5), Nos2 (2.4)
Adhesion	Vcam1 (1.8), Lamc1 (1.8), Tns1 (1.8), Cd38 (1.8), Itgam (1.7), Itgax (1.9), Thbs1 (1.5), Thbs2 (2.2), Clec4a1 (2.1), Clec4a3 (2.2), Antxr (2.6)
Angiogenesis	Ecm1 (2.1), Ptafr (1.6), Hpse (2.1), Atxr1 (2.6), Fgfr1 (1.6), Mmp13 (4.1), Mmp19 (1.7), Thbs1 (1.5), Nrp2 (1.8), Nrp1 (2.4), Crim1 (1.5), Ace (2.1), Plxdc2 (1.8)

C57BL/6 mice (4 mice per group) were challenged s.c. on the right limb with 3 × 10^5^ syngenic E.G7-OVA tumor cells. Mice were treated starting at day 13 with local radiation of 6 Gy applied in 3 equal doses on 3 consecutive days. Untreated mice served as controls. After 7 days tumors were exercised, RNA was isolated and gene expression was analyzed. Differentially expressed genes were identified by statistical analysis with the p ≤ 0.05 and a fold change of at least 1.5. The exact fold change is presented in the brackets.

**Table 2:** **Genes upregulated upon vaccination**

Pathway/function	Genes upregulated upon vaccination
**T cell signaling**	Cd8b1 (2.4), Cd8a (2.3), Il12r (1.8), Il2r (1.6), Ifng (1.6), Gzmb (3.0), Gzmk (1.7), Prf1 (2.0), Icos (2.1), Nkg7 (3.5), Stat1 (1.6), Stat4 (1.8)
**NK cell signaling**	Klrd1 (2.4), Klrk1 (1.7), Klrc1 (2.9), Nkg7 (3.5), Il12r (1.8), Gzmb (3.0), Gzmk (1.7), Prf1 (2.0), Fcgr3 (1.5), Fcgr4 (1.9)
**Chemotaxis**	Ccl3 (1.8), Ccl5 (2.7), Cxcl9 (1.9), Cxcl12 (1.5), Cxcl16 (2.2), Ccr5 (1.6)
**TLR signaling**	Tlr7 (2.2), Tlr8 (2.3), Tlr13 (1.8)
**MHC class II presentation**	H2-Dma (1.6), H2-Eb2 (1.7), H2-Aa (1.8), Cd74 (1.6), Cathepsins: k,s,c,d,h,l,w,a (1.5-2.1), Lgmn (1.8)
**Complement system**	C1qa (1.9), C1qb (1.7), C1qc (2.1), C1r (1.9), C1s (2.7), C2 (2.6), C3 (2.1), C3ar1 (1.7), Cfb (2.7)
**IFN-γ inducible GTPases**	Gbp2 (2.2), Gbp3 (1.8), Gbp4 (1.8), Gbp5 (2.0), Gbp6 (1.7)

C57BL/6 mice (4 mice per group) were challenged s.c. on the right limb with 3 × 10^5^ syngenic E.G7-OVA tumor cells. Mice were treated starting at day 13 with three vaccinations of OVA-encoded mRNA (32 μg/mouse, 2x week). Untreated mice served as controls. After 7 days tumors were exercised, RNA was isolated and gene expression was analyzed. Differentially expressed genes were identified by statistical analysis with the p ≤ 0.05 and a fold change of at least 1.5. The exact fold change is presented in the brackets.

**Table 3:** **Genes differentially regulated upon combination therapy in comparison to vaccinated mice**

Pathway/function	Downregulated genes
**Genes associated with tumor invasiveness**	Adam18 (6.7), Adam9 (1.6), Tgfbi (1.7), Mmp12 (1.8), Mmp3 (2.0), Mmp10 (2.2), Slc7a2 (2.4)
**Tumor associated factors**	Ly6i (3.1), Lrrc15 (2.4), Mamdc2 (2.4), Tm2d2 (2.1), Gpr97 (2.3), Grem1 (2.5), Dok4 (2.4), Tacc1 (2.3), Inhba (2.0), Ifitm1 (2.4)
**Pathway/function**	**Upregulated genes**
**Genes associated with tumor suppresion**	Phlda1 (2.3), Cxcl14 (2.4), Cd209a (2.4), Timp1 (1.9), Sfrp4 (3.9), Sulf1 (1.8), Nr1d1 (1.8), Ogn (5.0), Scara3 (2.3), Dpt (5.0)

C57BL/6 mice (4 mice per group) were challenged subcutaneously on the right limb with 3 × 10^5^ syngenic E.G7-OVA tumor cells. Mice were treated with the radioimmunotherapy starting at day 13 (local radiation of 6 Gy applied in 3 equal doses on 3 consecutive days and three vaccination with OVA-encoded mRNA – 32 μg, 2x week). OVA-encoded mRNA vaccinated mice served as controls. After 7 days tumors were exercised, RNA was isolated and gene expression was analyzed. Differentially expressed genes were identified by statistical analysis with the p ≤ 0.05 and a fold change of at least 1.5. The exact fold change is presented in the brackets.

### Analysis of LLC tumors reveals an improved infiltration of immune cells following combination therapy

**Figure 4:**
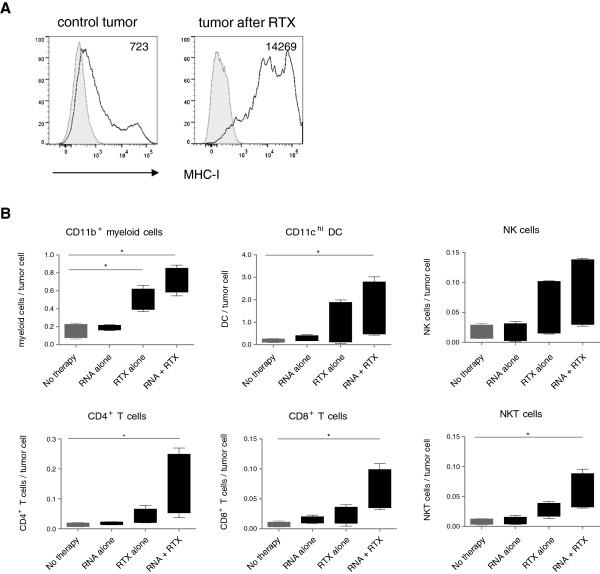
**Infiltration of immune cells into the large established immunosuppressive LLC tumors is improved after combination therapy.** C57BL/6 mice (n = 4 per group) were challenged subcutaneously on the right hind limb with 5 × 10^5^ 3LL-GFP cells. 18 days after tumor challenge mice were treated with local radiation alone (36 Gy total, divided into 3 equal fractions on 3 consecutive days) (RTX alone), vaccination alone (32 μg, twice a week, started at day 18) (RNA alone) or with radioimmunotherapy (with first vaccination given on day 19) (RNA + RTX). Untreated mice served as a control (No therapy). On day 25 tumors were excised, homogenized and immune cell infiltration analyzed by flow cytometry. **(A)** MHC class I expression was analyzed on single cell suspension of untreated- and irradiated tumor cells. **(B)** CD45.2^+^ host-derived immune cells were characterized by cell surface staining of several lineage markers and the ratio of different CD45.2^+^ immune cell subpopulations to CD45.2^-^ tumor cells is shown as median of four animals per group. * p < 0.05. Presented data show representative results of two independent analyses.

Gene expression analysis revealed a number of substantial and specific changes at the tumor sites following different therapies. Next, we asked whether these changes are reflected in the cellular composition of the tumor tissue. To obtain this information, we analyzed LLC tumor infiltrates by flow cytometry. LLC tumor cells were transplanted subcutaneously into the right limb and mice were left untreated until the tumor reached a volume of about 50 mm^3^. Tumors of the radiation (RTX alone) and the combined radioimmunotherapy (RNA + RTX) groups were irradiated with a total dose of 36 Gy, administered in 3 fractions of 12 Gy each on three consecutive days. Mice in the immunotherapy (RNA alone) and the radioimmunotherapy groups received two vaccinations per week. Untreated mice served as controls (No therapy). On day 25, tumors were isolated and the cellular composition was analyzed. At this time point, tumors did not differ significantly in volume (median volume was about 150-200 mm^3^). As shown in Figure 4A, irradiation of LLC tumors led to a strong upregulation of the MHC class I molecule. Additionally, the cellular composition of the tumor microenvironment was dramatically changed after irradiation and combination therapy compared to the untreated control tumors. As shown in Figure 4B when different populations of the tumor infiltrating immune cells were analyzed, we observed that tumor radiation induced a strong increase of myeloid and dendritic cells which are associated with activation of innate immune system. This was also the case for the combination therapy. Importantly, compared to control mice tumors from mice treated with combined immunoradiotherapy exhibited statistically significant increase in infiltration of CD4^+^, CD8^+^ T cells and NKT cells which are associated with the adaptive immune system. Despite the late onset of the immunotherapy also after vaccination alone improved infiltration of CD8^+^ T cells (fold change 1.4) and CD4^+^ T cells (fold change 0.6) could be observed suggesting beneficial effects of the immunotherapy even with the high tumor burden. The frequency of B cells was very low and levels did not change after the various treatments (data not shown). Taken together, our results show that local radiation of large established tumors results in cellular changes in the tumor microenvironment, making the tumor more accessible to mRNA-based immunotherapy.

## Discussion

Since the initial discovery of the tumor associated antigens (TAAs), which make cancer cells a potential target of the immune system, the efforts has been focused on designing therapies to induce potent and specific anti-tumor immune responses. This, together with the increased knowledge of the tumor biology, has resulted in the substantial progress made in recent years and has enabled immunotherapy to become promising strategy for cancer treatment [11]. An increasing amount of preclinical and clinical data underlines the therapeutic potential of combined anti-cancer approaches (reviewed in [12]). Here, we showed that two-component mRNA vaccines can be combined with local tumor irradiation to give a strong synergistic anti-tumor effect, even against low immunogenic LLC tumors. 3LL-GFP cell line used in our studies was characterized by an aggressive tumor growth suggesting lack of the rejecting responses generated against GFP protein. This stays in agreement with data provided by Skelton et al. showing minimal immunogenicity of GFP in C57BL/6 mice contrary to Balb/c strain [13]. In addition, we demonstrated that combination therapy results in the complete eradication of large established E.G7-OVA tumors with a size of ca. 150 mm^3^ in a significant number of mice for which monotherapies were mostly ineffective. The remaining mice exhibited a delayed tumor growth. In our quest to better understand the synergistic effects of radioimmunotherapy we analyzed the effects of radiation on the gene expression in comparison to mice which received vaccination only. Combination therapy showed the upregulation of some interesting genes coding for tumor suppressor proteins. Concomitantly, genes coding for the proteins described as tumor associated factors or molecules promoting tumor invasion were downregulated. The flow cytometric analysis of tumors treated with combination therapy versus monotherapy, revealed an increased infiltration of cells associated with the activation of the adaptive immune system. LLC tumors are known for their strong immunosuppressive properties; therefore immunotherapy of already established tumors with the volume between 50-100 mm^3^ cannot inhibit the tumor escape. Notably, the activation of immune system has occurred as demonstrated by the improved CD8^+^ T cell infiltration (compared to the control mice) followed the vaccination alone. Interestingly, vaccination alone tended to decrease frequency of regulatory T cells (Tregs) within the tumor whereas radiation exhibited the opposite effect as previously shown [14]. More importantly, this increase was not detectable in the combination treatment group further suggesting beneficial effect of the vaccination (Additional file 1: Figure S2). Taken together, our data provide evidence that the combination of immunotherapy with local tumor irradiation creates an immune response which results in efficient tumor treatment. mRNA vaccines in combination with irradiation, generate a different immune response compared to the monotherapies alone. This can be seen in the gene expression profile of tumors treated with the combination therapy, which show a completely different gene expression pattern to the monotherapies. The downregulation of peptidases, responsible for the degradation of extracellular matrix and for tumor invasion, was only observed in mice treated with the combination therapy. Interestingly, some genes coding for tumor suppressors were only upregulated in the group treated with radioimmunotherapy. The secreted frizzled-related protein 4 (SFRP4), proposed as a tumor suppressor in many cancers based on its loss in patients’ tumors [15], was upregulated about 4 fold in mice treated with the combination therapy. Jacob et al. reported that the loss of SFRP4 correlates with an aggressive phenotype and predicts poor outcome in ovarian cancer patients [16]. Moreover they postulated a role for SFRP4 as a tumor suppressor gene in ovarian cancers via inhibition of the Wnt signaling pathway. Dermatopontin (DPT), another gene strongly upregulated by the combination therapy, codes for a tumor suppressor. Li and colleagues showed that DPT is expressed in human liver and is significantly downregulated in hepatocellular carcinoma. Its loss is postulated to be associated with carcinogenesis [17]. Recent work from Yamatoji et al. described DPT as a potential predictor for metastasis of human oral cancer [18]. They showed that DPT expression in primary oral squamous cell carcinoma cells was significantly lower than in normal counterparts and correlated with regional lymph node metastasis. The authors postulated that downregulation of DPT is a characteristic event in oral squamous cell carcinoma and that this protein might play an important role in regulating tumor invasion and metastasis [18]. We also found that the osteoglycin gene (ogn) was upregulated 4.6 fold in mice treated with the combination therapy. Its downregulation is also described as a diagnostic protein marker in squamous cervical cancer [19]. In summary, the strong upregulation of tumor suppressor genes found in the group treated with immunotherapy and radiation underlines the clinical relevance and therapeutic potential of the combination therapy.

The increased frequency of mice which survived E.G7-OVA tumor challenge in the combination group compared to the monotherapy groups is another indication of the distinct immune response induced by mRNA vaccine/irradiation therapy. Furthermore, majority of mice that remained tumor-free after E.G7-OVA tumor challenge survived subsequent challenge with the parental EL-4. This suggests a possible induction of memory T cells, which are able to recognize other tumor-associated antigens besides the specific one used in the vaccination. The induction of broad immune protection against antigen-negative tumors is highly desirable and can be achieved via induction of epitope spreading. Reits et al. showed in the murine colon adenocarcinoma that radiation can modulate the peptide repertoire, by expanding the intracellular peptide pool [3]. Additionally, Nesslinger et al. tested a poxvirus-based vaccine encoding prostate-specific antigen (PSA) with radiotherapy in patients with localized prostate cancer. They could show that vaccination against PSA, combined with external beam radiation therapy, induced immune responses to additional tumor-associated antigens. The epitope spreading was observed in a large number of patients treated with vaccine and radiation [20]. Similar results were obtained by Gully and colleagues in a phase II clinical trial. They observed that the combination of a poxviral vaccine encoding PSA with radiotherapy, induced not only a PSA-specific T-cell response in patients with clinically localized prostate cancer, but also showed evidence of *de novo* generation of T cells with specificity for different antigens [21]. With these encouraging results, further clinical trials have been initiated to test the safety and clinical efficacy of radioimmunotherapy (e.g. clinical trial NCT00450619 – testing combination of Prostvac with radiotherapeutic Quadramet in prostate cancer or clinical trial NCT00085241, in which CEA-TRICOM was combined with local radiation of liver metastases). Also at the preclinical stage, the positive impact of radiation together with vaccination/anti-CD25 mAb regimen in the elimination of established tumors was shown, suggesting a possible broad application of this combination therapy [22].

An important role of ionizing radiation is its ability to modify the tumor microenvironment, thus limiting immunosuppressive capacity and enabling efficient homing of immune cells to the tumor [3]. It has been previously shown that CXCL16 is responsible for attracting tumor-specific T cells to the irradiated tumor site [23] and is a crucial component of the successful combination of radiotherapy and anti-CTLA4 treatment in a mouse breast cancer model [24]. In our study we also observed the induction of CXCL16 following radiation therapy (Table 1). Tumor vasculature is characterized by downregulation of adhesion molecules which decreases the ability of T cells to home to the tumor site [25]. Our data show that radiation can increase the expression of vascular cell adhesion molecule (VCAM)-1, a phenomenon which was also observed by others [26]. This could also contribute to the enhanced trafficking of immune cells to the tumor site and their retention there. In addition, we observed increased infiltration of CD11b^+^ and CD11c^+^ cells following both irradiation and the combined treatment (Figure 4). The role of macrophages, the biggest cell subset expressing CD11b marker, in the immune response is controversial. However, Kawai et al. showed that an increase in these cells in cancer nests of NSCLC after chemotherapy is a positive prognostic predictor of patients’ survival [27]. Of note, further characterization of the tumor-infiltrating myeloid cells revealed increased frequencies of myeloid derived suppressor cells (MDSCs) following radiation treatment as previously described [28]. However, addition of vaccination did not further enhance this population with vaccination alone having a beneficial effect on the numbers of MDSCs within the tumor (Additional file 1: Figure S3). In addition, both macrophages and CD11c^+^ dendritic cells are responsible for the clearance of irradiation-induced dead or damaged tissues followed by antigen presentation and T cell activation [29, 30]. It is therefore of interest that both radiation and the combination therapy increased tumor infiltration by cross-presenting CD8^+^ DCs (Additional file 1: Figure S4). A positive correlation between improved survival and intratumoral dendritic cells (DCs) has been shown in hepatocellular carcinoma [31] and several lines of evidence demonstrate DCs participation in anti-tumor immunity [32]. Moreover, both microarray and flow cytometric data indicated an increase in CD4^+^ and CD8^+^ T cells as well as NK cell infiltration. In accordance to the presented data, a positive correlation between tumor-infiltrating T cells and favorable prognosis has been described in a variety of different cancer types such as hepatocellular carcinoma [33], pancreatic carcinoma [34, 35], lung carcinoma [36], colorectal cancer [37, 38] and melanoma [39].

In conclusion, our results demonstrate that tumor-specific mRNA-based vaccines can be effectively combined with standard cancer therapies, such as radiation to create highly synergistic anti-tumor effects. We strongly believe that the combination approaches will play central role in the future clinical developments, opening the possibility to attack tumors via complementary, synergistically acting mechanisms and consequently improve long-term survival of cancer patients.

## Electronic supplementary material

Additional file 1: Figure S1: LLC tumor growth after various radiation doses. **Figure S2.** Infiltration of Lewis Lung Carcinoma tumors by Tregs following various treatments. **Figure S3.** Infiltration of Lewis Lung Carcinoma tumors by MDSCs following various treatments. **Figure S4.** Infiltration of Lewis Lung Carcinoma tumors by CD8^+^ DCs following various treatments. **Figure S5.** Gating strategy for analyzing tumor infiltrating DCs. (PDF 360 KB)
