# Supplementary material for: mRNA-based vaccines synergize with radiation therapy to eradicate established tumors
Source: Radiat Oncol. 2014 Aug 15;9:180. doi: 10.1186/1748-717X-9-180 (PMC4150951; doi:10.1186/1748-717X-9-180)
Supplement: Supplementary file 1 — Additional file 1: Figure S1: LLC tumor growth after various radiation doses. Figure S2. Infiltration of Lewis Lung Carcinoma tumors by Tregs following various treatments. Figure S3. Infiltration of Lewis Lung Carcinoma tumors by MDSCs following various treatments. Figure S4. Infiltration of Lewis Lung Carcinoma tumors by CD8+ DCs following various treatments. Figure S5. Gating strategy for analyzing tumor infiltrating DCs. (PDF 360 KB) [file 13014_2014_1150_MOESM1_ESM.pdf]

Supplementary materials

mRNA-based vaccines synergize with radiation therapy to eradicate established tumors

Mariola Fotin-Mleczek, Kai Zanzinger, Regina Heidenreich, Christina Lorenz, Aleksandra Kowalczyk, Karl-Josef Kallen and Stephan M. Huber

Figure 1. LLC tumor growth after various radiation doses.

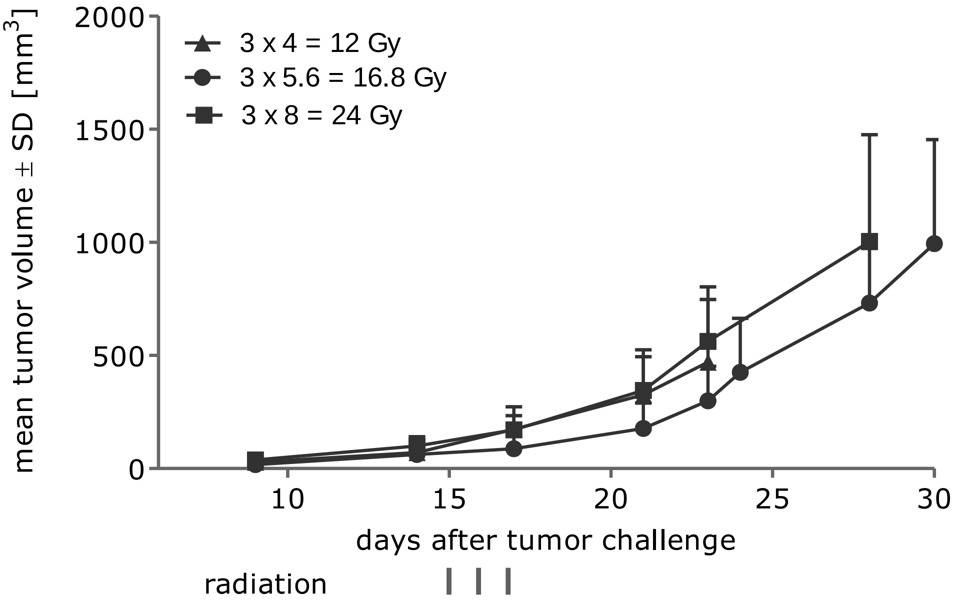

C57BL/6 mice (10 per group) were challenged subcutaneously on the right limb with 0.1x10 LLC cells. Mice were divided into three groups and locally irradiated with the radiation dosage (12, 16.8 or 24 Gy) applied in equal doses on three consecutive days starting on day 15.

Figure 2. Infiltration of Lewis Lung Carcinoma tumors by Tregs following various treatments.

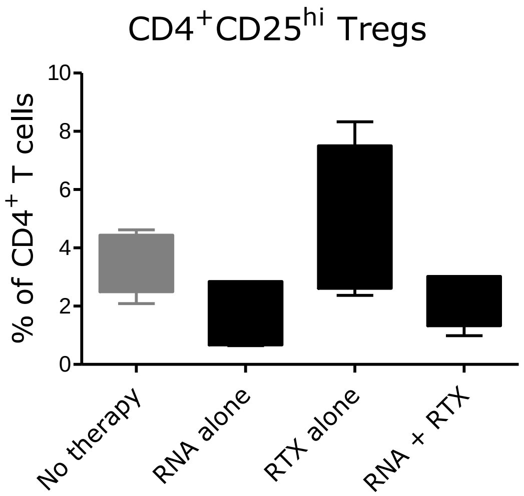

C57BL/6 mice (n = 4 per group) were challenged s.c. on the right hind limb with $5x10^5$ 3LL-GFP cells. 18 days after tumor challenge mice were treated with local radiation alone (36 Gy total, divided into 3 equal fractions on 3 consecutive days) (RTX alone), vaccination alone (32µg, twice a week, started at day 18) (RNA alone) or with radioimmunotherapy (with first vaccination given on day 19) (RNA+RTX). Untreated mice served as a control (No therapy). On day 25 tumors were excised, homogenized and immune cell infiltration analyzed by flow cytometry. Tregs were characterized as $CD45.2^+CD3^+CD4^+CD25^{hi}$ cells. Frequency of Tregs within $CD4^+$ T cells is shown as median of four animals per group.

Figure 3. Infiltration of Lewis Lung Carcinoma tumors by MDSCs following various treatments.

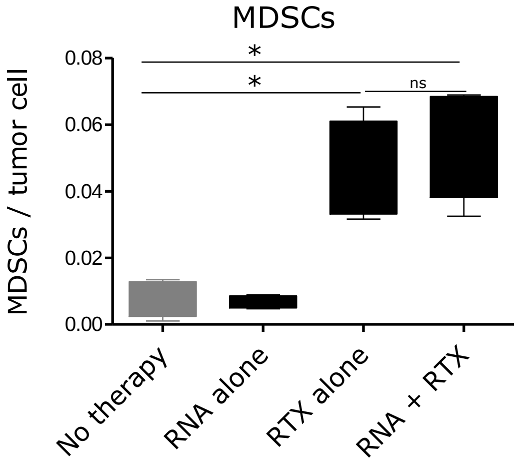

C57BL/6 mice (n = 4 per group) were challenged s.c. on the right hind limb with $5x10^5$ 3LL-GFP cells. 18 days after tumor challenge mice were treated with local radiation alone (36 Gy total, divided into 3 equal fractions on 3 consecutive days) (RTX alone), vaccination alone (32µg, twice a week, started at day 18) (RNA alone) or with radioimmunotherapy (with first vaccination given on day 19) (RNA+RTX). Untreated mice served as a control (No therapy). On day 25 tumors were excised, homogenized and immune cell infiltration analyzed by flow cytometry. MDSCs were characterized as $CD45.2^+CD11b^+Gr\text{-}1^{hi}$ cells and the ratio of MDSCs subpopulation to $CD45.2^-$ tumor cells is shown as median of four animals per group. *$p<0.05$

Figure 4. Infiltration of Lewis Lung Carcinoma tumors by $CD8^+$ DCs following various treatments.

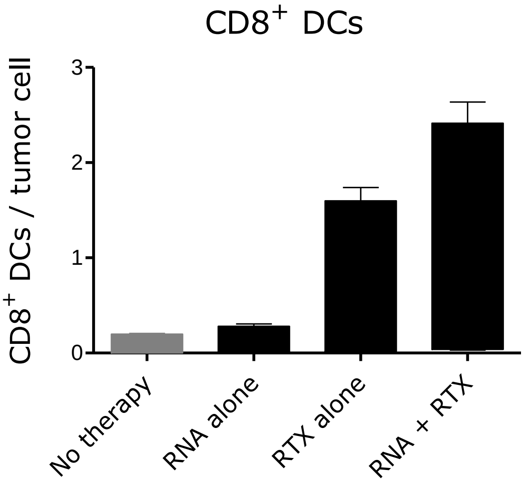

C57BL/6 mice (n = 4 per group) were challenged s.c. on the right hind limb with $5x10^5$ 3LL-GFP cells. 18 days after tumor challenge mice were treated with local radiation alone (36 Gy total, divided into 3 equal fractions on 3 consecutive days) (RTX alone), vaccination alone (32µg, twice a week, started at day 18) (RNA alone) or with radioimmunotherapy (with first vaccination given on day 19) (RNA+RTX). Untreated mice served as a control (No therapy). On day 25 tumors were excised, homogenized and immune cell infiltration analyzed by flow cytometry. $CD8^+$ DCs were characterized as $CD45.2^+CD11c^+CD8^+$ cells and the ratio of $CD8^+$ DCs subpopulation to $CD45.2^-$ tumor cells is shown as median of four animals per group.

Figure 5. Gating strategy for analyzing tumor infiltrating DCs.

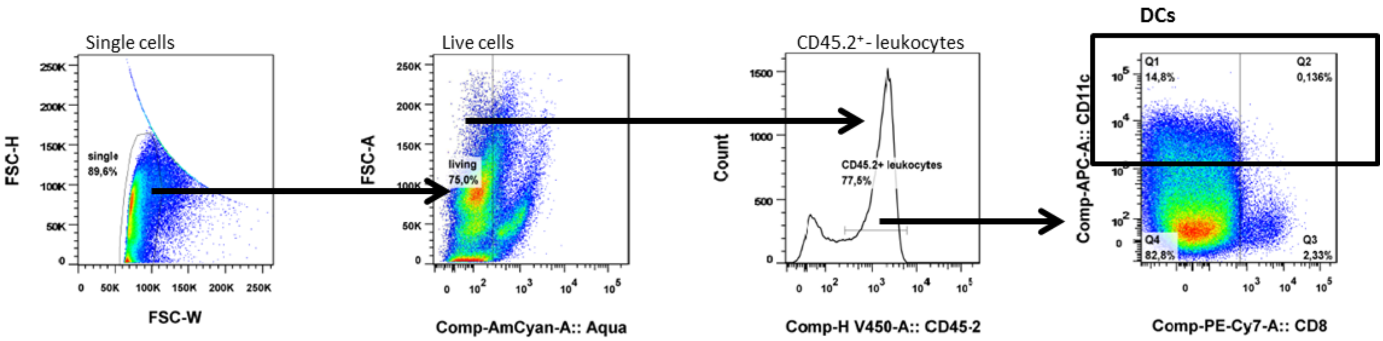

Mice were treated as described in the figure legend to Figure 3 of the main manuscript and the gating strategy to identify DCs is shown. Cells were first gated on single cells to exclude doublets, follow by live/dead staining and CD45.2 staining for lymphocytes. DCs were characterized as $CD11c^{+}$ within lymphocyte gate ($CD45.2^{+}$) (including $CD8^{+}$ and $CD8^{-}$ cells) (as depicted by the rectangular box).
